# Non-Invasive Ventilation Therapy Implementation in Medical Wards—A Scoping Review to Understand Hospitals’ Protocols and Procedures

**DOI:** 10.3390/jcm14228152

**Published:** 2025-11-17

**Authors:** Catherine Buchan, Idinakachukwu Chukwu, Anne Walker, Eli Dabscheck, Natasha Smallwood

**Affiliations:** 1 Department of Respiratory Medicine, The Alfred Health, 55 Commercial Road, Melbourne, VIC 3004, Australia or c.buchan@alfred.org.au (C.B.);; 2 Respiratory Research@Alfred, School of Translational Medicine, The Alfred Centre, Monash University, Melbourne, VIC 3004, Australia; 3 Department of Respiratory and Sleep, Central Adelaide Local Health Network, Adelaide, SA 5000, Australia; 4 Faculty of Health and Medical Sciences, University of Adelaide, Adelaide, SA 5000, Australia

**Keywords:** non-invasive ventilation, local health policy and guidance, acute respiratory failure, ward-delivered

## Abstract

**Background/Objectives**: Non-invasive ventilation (NIV) is a highly effective and safe treatment for hypercapnic acute respiratory failure (ARF). This study aimed to identify and examine the content and recommendations within local health guidance documents (LHGDs) that facilitate ward-based implementation of NIV for the treatment of ARF in adults in Australian health services. **Methods**: A scoping review was conducted on 26 May 2023, updated on 11 January 2024 and 16 September 2025 to identify public health services NIV LHGDs. Data were extracted and analysed regarding NIV initiation, monitoring, maintenance and weaning, and management of clinical deterioration. **Results**: Twenty-two LHGDs were included, of which only six (27.3%) referenced international NIV guidelines. Most LHGDs (n = 17, 77.3%) required an arterial blood gas (ABG) measurement before NIV initiation, with eight (36.4%) specifying PaCO_2_ > 45 mmHg and pH < 7.35 as a basis to consider NIV initiation. Most (n = 13, 59.1%) specified a target SpO_2_ range to monitor NIV, but recommendations varied. NIV implementation recommendations general wards (n = 12, 54.5%) were most common, followed by respiratory wards (n = 5, 22.7%) and respiratory care units (n = 4, 18.2%). Most LHGDs did not specify criteria for medical review (n = 13, 59.1%), clinical escalation (n = 13, 59.1%) or palliation care (n = 13, 59.1%), and weaning guidance was rarely specified (n = 7, 31.8%). **Conclusions**: There was substantial variation in the structure and content of LHGDs for ward-based NIV across Australian hospitals, including inconsistencies in initiation, monitoring, weaning, and detection of patient deterioration. Variation and limited alignment with major clinical guidelines may impact care quality and safety.

## 1. Introduction

Acute respiratory failure (ARF) is a common and serious medical condition, making it the leading cause of hospitalisation with high morbidity and mortality [1,2]. ARF is characterised by ventilatory failure that leads to inadequate oxygenation (hypoxemia), with or without carbon dioxide retention (hypercapnia). Conventional oxygen therapy (COT), high-flow nasal oxygen (HFNO), and non-invasive ventilation (NIV) are forms of non-invasive respiratory support (NIRS) that can be safely delivered to adults with ARF in medical wards, outside of intensive care units and emergency departments [3,4,5]. For more than two decades, ward-delivered NIV has been used across various settings (e.g., medical and respiratory wards, and respiratory care units) and, when delivered by a multidisciplinary respiratory team, has proven to be a safe and effective treatment [5,6,7,8,9,10]. A 2024 systematic review found that prompt use of NIV in acute COPD exacerbations is highly effective and safe, significantly reducing mortality, intubation rates, complications, and hospital stay compared to invasive mechanical ventilation [11].

Real-world international surveys regarding acute NIV have highlighted significant variations in clinical practice compared to evidence from randomised controlled trials and established guideline recommendations [12,13,14,15]. These surveys reveal substantial variation in NIV implementation, including differences in clinical indications, ventilation and oxygen parameters, models of care (intensive care unit (ICU), respiratory, and general wards), the range of healthcare professionals involved (doctors, nurses, physiotherapists), availability of monitoring, organisational resources, and level of clinician education and training [12,13,14,15]. Moreover, reported real-world NIV success rates are lower than those achieved in trials, with delays in NIV initiation leading to higher failure rates [14] and uncertain efficacy for complex patients, particularly in settings with less access to monitoring [13,16].

In 2014, a Danish single-site hospital conducted an audit to assess the safety of NIV delivery, revealing that guideline-concordant care is achievable, with 82.3% of COPD admissions to the respiratory ward receiving appropriate NIV treatment [17]. However, in 2015, a Canadian study, including 11 hospitals and 330 patients, found significant variations in NIV provision and mortality (*p* = 0.006) [18]. More recently, the British Thoracic Society (BTS) national NIV report (2019), which included 158 UK hospitals and 3502 episodes of patient care, reported significant variation in NIV implementation approaches (models of care, patient selection and time to treatment, clinical workforce and organisational resources) and patient outcomes [19,20].

Clinical practice guidelines strongly recommend NIV as the first-line treatment for acute hypercapnic respiratory failure in patients with COPD exacerbations [21,22]. However, NIV is not advised as first-line therapy for all respiratory conditions presenting with acute hypercapnic respiratory failure, such as asthma exacerbations [21,23]. A crucial requirement for the safe and consistent implementation of NIV in hospitals is the development of local health guidance documents (LHGDs), including policies and protocols based on national and international clinical practice guidelines. These documents outline the practical steps for NIV delivery, staff training and ongoing local audits to monitor clinical practice and patient outcomes [11,13,23,24,25,26]. In the absence of clear local guidance, NIV tends to be used less frequently and inconsistently across hospital settings [11,13]. However, the availability and content quality of LHGDs is not known.

This study aimed to identify and examine the content and recommendations within LHGDs that facilitate ward-delivered NIV for the treatment of ARF in adults being cared for in Australian hospitals. Additionally, we aimed to assess the alignment of LHGDs with national and international clinical guidelines. We hypothesised that healthcare organisations’ LHGDs would vary widely in content and structure. Additionally, we aimed to develop a core set of standardised information regarding NIV implementation to inform LHGDs nationally.

## 2. Materials and Methods

A scoping review was conducted to identify and examine LHGDs from public hospitals detailing the implementation of NIV in medical wards for patients with ARF in Australia. This methodical approach maps relevant content beyond peer-reviewed literature to identify potential gaps [27]. The scoping review protocol was registered on the Open Science Framework (https://osf.io/ztkjp, accessed on 17 July 2024). The “PROMPT” database and South Australian local health network databases were searched on 26 May 2023, updated on 11 January 2024 and updated on 16 September 2025. The PROMPT database (https://prompt.org.au/, accessed on 29 June 2024) is an online guidance document management system for 110 public and private health services in Victoria and South Australia. It aims to facilitate sharing of LHGDs between participating health services in order to support safe and high-quality clinical care. Databases were searched using the words “non-invasive ventilation”, which identified documents that included keywords alone or in combination. LHGDs from healthcare organisations affiliated with the study team, but not using PROMPT, were also eligible for inclusion. The core set of standardised NIV implementation information will be developed following review of the LHGDs and synthesis of recommendations, and alignment of these recommendations with current clinical practice guidelines based on the expert opinion of the authors.

### 2.1. Inclusion and Exclusion Criteria

LHGDs identified from the search were included if they met the following criteria: the document was labelled as a policy, procedure, protocol, local guideline, or a combination of any of these; the title contained the term “non-invasive ventilation” or similar; the content focused on ward-delivered NIV implementation for adults with ARF. Where multiple documents existed from a health service, all were included if they met the inclusion criteria. LHGDs were excluded if they focused only on NIV delivery in aged care, palliative care, intensive care, emergency care or home settings.

### 2.2. Data Screening, Extraction and Analysis

Two independent reviewers (MF and AC) conducted title and full-text screening, with differences resolved by discussion among the study team (NS, CB, and AW). Data were extracted using a specifically developed data extraction tool, with key domains including: (a) indications for NIV, (b) NIV initiation, monitoring, maintenance and weaning, and (c) processes for identifying clinical deterioration and escalating care. Two reviewers (CB and IC) independently extracted and then cross-checked the data. Data are reported using descriptive statistics (Microsoft Excel, v16.63.1).

## 3. Results

**Figure 1:**
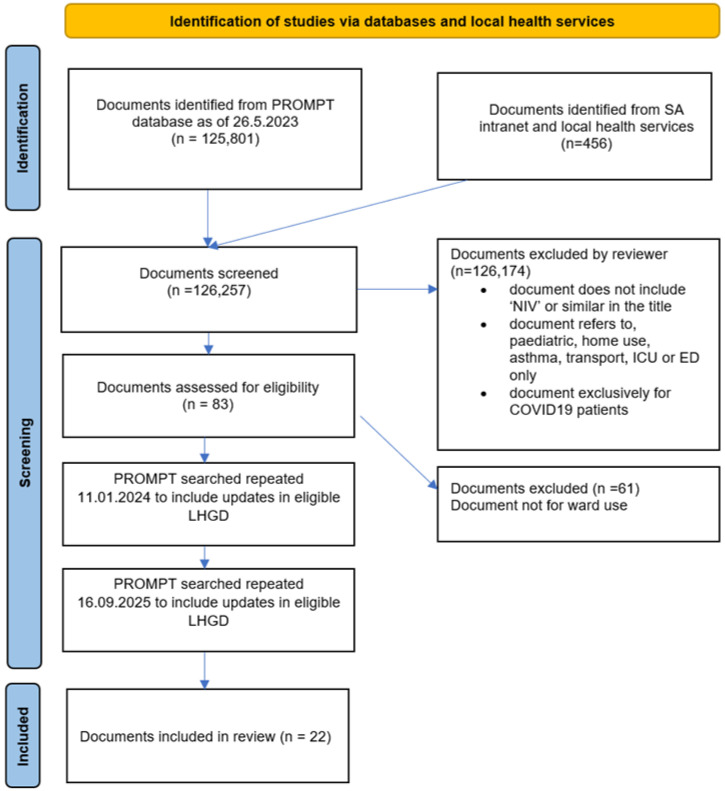
PRIMA diagram.

The search of PROMPT detected 125,801 documents, with an additional 456 documents identified from South Australian Health services. Twenty-two documents met the inclusion criteria and were included in this study. No new or amended LHGDs were identified during the updated search on 11 January 2024, and the subsequent updated search on 16 September 2025 identified 17 documents as unchanged, while six had updated version numbers, including one with a revised title but no substantive content changes. Additionally, three documents had been modified to limit implementation support provided (Figure 1).

### 3.1. Characteristics of Included Documents

Of 22 LHGDs, most (n = 17, 77.3%) were from Victorian health services and over half (n = 13, 59.1%) were from metropolitan hospitals, with seven documents from rural (31.8%) and two from regional (9.1%) hospitals. The LHGD types comprised local guidelines (n = 12, 54.5%), procedures (n = 7, 31.8%) and policies (n = 3, 13.6%). The most frequently specified target audience for the documents were nurses (n = 10, 45.5%), followed by doctors (n = 9, 40.9%) and physiotherapists (n = 4, 18.2%). Nine (40.9%) documents did not specify a target audience, with five (22.7%) reporting being relevant for “all clinical staff”. The LHGDs that listed more than one clinical discipline as the target audience (i.e., nurses and doctors) did not specify which discipline (medical, nursing or physiotherapy) was responsible for patient reassessment after NIV initiation.

International clinical practice guidelines for NIV use to treat hypercapnic ARF were rarely referenced. Four documents (18.2%) referenced the 2016 BTS/Intensive Care Society (ICS) guideline for NIV management of adults with hypercapnic ARF [23]. Two LHGD (9.1%) referenced the 2017 European Respiratory Society/American Thoracic Society (ERS/ATS) acute NIV guideline for the management of ARF [21]. One document (4.5%) referenced the Australian State of New South Wales Agency of Clinical Innovationߣs acute NIV clinical practice guidelines from 2014 [28]. None of the LHGDs referenced the 2015 Thoracic Society of Australia and New Zealand (TSANZ) Acute Oxygen Guidelines [29], the updated 2021 TSANZ Acute Oxygen Position Statement [30], or the 2022 ERS clinical practice guidelines for high-flow oxygen, which included recommendations for NIV and HFNO [31].

Seven documents (n = 31.8%) included HFNO recommendations in NIV LHGDs; however, they lacked specific recommendations on safe initiation, monitoring, and weaning of HFNO. One document (4.5%) recommended HFNO as the preferred modality for weaning from NIV.

### 3.2. Considerations for NIV Initiation

All LHGDs provided indications for using NIV, with the most frequent being for the management of an acute exacerbation of COPD with hypercapnic ARF (n = 16, 72.7%) and acute pulmonary oedema (n = 12, 54.5%) (Table 1). Eleven documents (50%) included a definition for hypercapnia based on the ABG, partial pressure of carbon dioxide (PaCO_2_). Of these, eight (36.4%) recommended PaCO_2_ > 45 mmHg together with pH < 7.35 as a basis to consider NIV initiation (Table 1).

The majority of LHGDs (n = 19, 86.4%) described contraindications to NIV implementation, with the most frequently cited being imminent cardiac or respiratory arrest (n = 20, 90.9%), a person’s inability to protect their own airway or manage secretions (n = 17, 77.3%), untreated pneumothorax (n = 16, 72.7%), facial injury or burns (n = 15, 68.2%) and altered conscious state (n = 9, 40.9%) (Supplementary Table S1). Less than half of the LHGDs (n = 9, 40.9%) recommended reviewing patient goals of care when initiating NIV. The majority (n = 17, 77.3%) advised communicating with the patient the need to initiate NIV therapy; however, none provided guidance on communicating with non-English speaking patients in these situations (Table 1).

### 3.3. NIV Initiation and Maintenance Recommendations

Most LHGDs (n = 17, 77.3%) required an ABG to have been measured before NIV initiation. Three (13.6%) documents recommended venous blood gas (VBG) measurement as a suitable alternative to assess carbon dioxide levels before NIV implementation. Almost half of the documents (n = 9) specified a target SpO_2_ range at NIV initiation with or without co-prescribed oxygen, and most (n = 13, 59.1%) recommended SpO_2_ monitoring and documentation (Table 2). The SpO_2_ target level recommendations varied, with almost a third of LHGDs recommending the lower SpO_2_ level range of greater than 88% (n = 7) and the upper-level SpO_2_ range of greater than 92% (n = 7) (Table 1). The LHGDs did not include recommendations on ventilation modes, inspiratory time or cycling parameters, nor specify tidal volume or minute ventilation targets. Recommendations regarding which physiological and ventilator observations should be undertaken whilst on NIV and the frequency varied (Table 2). None of the LHGDs included specific recommendations regarding monitoring work of breathing, respiratory distress or dyspnoea assessment.

Most LHGD recommended that NIV therapy should be initiated and prescribed by senior (n = 14, 63.6%) or junior (n = 3, 13.6%) doctors, with very few documents recommending initiation by senior nurses (n = 4, 18.2%) or senior physiotherapists (n = 1, 4.5%). Thirteen LHGD (59.1%) did not propose use of an NIV prescription form, with very few suggesting a digital (n = 5, 22.7%) or paper (n = 4, 18.2%) prescription. Recommendations for NIV initiation and maintenance prescription regarding ventilation parameters varied widely and did not include ventilation modes, inspiratory time or cycling parameters, nor specify tidal volume or minute ventilation targets. Registered nurses (n = 11, 50.0%) were most often described as physically applying NIV to patients, with some documents specifying specialist respiratory nurses 31.8% (n = 7), nurse consultants (n = 2, 9.1%), enrolled nurses (n = 1, 4.5%) or nurse practitioners (n= 1, 4.5%).

Just over half the LHGDs (n = 12, 54.5%) recommended NIV implementation on general wards, while few specified respiratory wards (n = 5, 22.7%) or respiratory care units (n = 4, 18.2%) as the setting for managing patients on NIV (Table 2). Most LHDGs recommended an initial registered nurse-to-patient ratio of one-to-one (n = 15, 68.2%); however, eight (36.4%) did not state the nurse-to-patient ratio required.

Most LHGDs (n = 14, 63.6%) highlighted potential adverse effects, including pressure sores (n = 14, 63.6%), aerophagia (n = 12, 54.5%), hypotension (n = 6, 27.3%) and barotrauma or pneumothorax (n = 6, 27.3%) (Supplementary Table S1). Twelve (54.5%) documents provided strategies to prevent pressure injuries; however, hydration and nutrition recommendations were rarely provided (n = 5, 22.7%).

### 3.4. Recognising and Addressing Clinical Deterioration

Maximum ventilation parameters (e.g., inspiratory and expiratory pressures) (n = 2, 9.1%), oxygen parameters (e.g., litres per minute) (n = 2, 9.1%) and types of ventilators (n = 14, 63.6%) that could be safely implemented in the ward setting were provided inconsistently. Nine LHGD (40.9%) specified criteria for review by medical staff (such as persistent tachypnoea or hypoxia), yet less than half (n = 9, 40.9%) specified criteria for escalation of care to an intensive care unit. Local early warning systems to recognise clinical deterioration were often not stated (n = 9, 40.9%). Among the LHGDs that included patient deterioration systems, the most common were clinical review criteria (n = 9, 40.9%), followed by escalation criteria for medical emergency team response (n = 8, 36.4%). Additionally, 9 documents (40.9%) outlined criteria for considering palliative care referral.

### 3.5. Weaning

Guidance to support weaning patients from NIV was rarely specified (n = 7, 31.8%), with clinical improvement (n = 5, 22.7%), improved work of breathing and respiratory rate (n = 4, 18.2%), normalised PaCO_2_ (n = 3, 13.6%) and normalized pH (n = 3, 13.6%) being the most recommended indications for weaning (Table 3). Nearly sixty percent (n = 13) of LHDGs specified target SpO_2_ ranges of 88–92% when weaning from NIV with or without prescribed oxygen. No LHGDs specified targets for ventilator parameters (e.g., inspiratory and expiratory pressures) or oxygen parameters (e.g., litres per minute) prior to considering weaning. Most did not specify (n = 15, 68.2%) guidance on the approach to weaning or the physiological parameters to monitor. When provided, the LHGD recommended a reduction in NIV hours during the day or the day and night (n = 5, 22.7%). Four (18.2%) documents recommended HFNO be used as a weaning aid.

Following the review of the LHGDs and their alignment with current international recommendations for NIV, the authors developed a guidance template based on expert opinion comprising essential information for implementing ward-based NIV in adults with ARF (Table 4) [21,23]. This core framework aims to standardise practice and enhance the quality of care in ward settings.

## 4. Discussion

To our knowledge, this scoping review is the first to examine hospital guidance supporting the implementation of ward-delivered acute NIV to adults with hypercapnic ARF. Information was insufficient or varied between LHGDs in several key areas, including definitions for hypercapnia, oxygen saturation targets, NIV prescription recommendations, NIV initiation, maintenance and weaning, and addressing clinical deterioration. These deficiencies and variations in recommendations could impact clinical decision-making and contribute to variations in patient care. Importantly, despite the absence of Australian NIV clinical practice guidelines, international guidelines were referenced rarely [21,23].

The BTS/ICS acute NIV clinical practice guideline recommends obtaining an ABG before NIV initiation to confirm acute respiratory acidosis (pH < 7.35 and PaCO_2_ > 48 mmHg) and on NIV commencement with controlled oxygen therapy to stipulate a target SpO_2_ range for people with COPD exacerbations of 88–92% and as a practice recommendation to be applied to all causes of hypercapnic ARF [23]. Similarly, the more recent ERS/ATS acute NIV clinical practice guideline recommends NIV use for people with COPD exacerbations and acute respiratory acidosis (pH < 7.35 and PaCO_2_ > 45 mmHg) with a high certainty of evidence [21]. However, half of the LHGDs lacked a definition for hypercapnia based on ABG parameters (pH and PaCO_2_), and when definitions were provided, most were inconsistent with international guidelines. Notably, although international guidelines [21] do not recommend NIV for hypercapnic ARF in people with asthma exacerbations, nearly a quarter of LHGDs listed this as an indication for acute NIV use [11,12]. This is particularly concerning, as ward-delivered NIV in asthma exacerbations carries a high risk of unrecognised treatment failure and clinical deterioration [33].

To treat hypercapnic ARF, controlled oxygen is often co-prescribed with NIV, with target oxygen saturations of 88–92% recommended in people with COPD and/or those suspected of being susceptible to oxygen-induced hypercapnia [23,30]. In our study, target SpO_2_ recommendations varied considerably across the LHGDs; while most advised a range of 88–92% during NIV for hypercapnic ARF, irrespective of co-prescribed oxygen, more than one-fifth of the documents recommended maintaining SpO_2_ above 94% without offering a clinical rationale to support this recommendation.

The lack of consistency in recommendations for controlled oxygen management in the setting of hypercapnic ARF is of clinical concern and potential practice implications, as liberal oxygen administration, resulting in SpO_2_ levels above 94–96%, has been associated with increased in-hospital mortality among acutely ill patients [34]. To reduce the clinical uncertainty of variable SpO_2_ targets for different conditions, and to mitigate the risks associated with over-oxygenation, utilisation of a consistent target SpO_2_ range of 88–92% is recommended for all cases of hypercapnic ARF receiving NIV, irrespective of whether supplemental oxygen is co-administered [23,35].

National and international clinical guidelines for oxygen and NIV recommend formal written prescriptions including target SpO_2_ ranges and titration of the therapies to treatment goals [21,23,30,32,36]. While Australia has a standardised national medication chart to ensure accurate and safe prescribing and drug administration in hospitals, a standardised prescription format for acute oxygen and/or NIV is lacking for hospitalised patients [36]. This may contribute to variable oxygen and NIV prescription practices in hospitals and patient harm [37,38,39,40,41]. Most LHDGs did not recommend either paper or digital NIV prescription; however, implementation of a standardised prescription for oxygen, NIV and other respiratory support systems (e.g., CPAP) could enhance clinical care quality and safety.

Despite over two decades of safe implementation of ward NIV treatment, failure may occur; thus, early recognition and timely escalation of care (e.g., to ICU for mechanical ventilation) or provision of palliative care are essential [5,7,8,22,42]. The GOLD 2025 report recommends immediate transfer to a respiratory care unit or ICU for advanced respiratory management if PaO_2_ remains low or pH falls below 7.25 despite NIV during an acute COPD exacerbation; however, the 2017 Cochrane systematic reviews concluded that the magnitude of benefit for NIV in reducing mortality is similar when NIV is applied on the ward or in the ICU [9,22]. The BTS/ICS NIV guideline recommends continuous monitoring combined with early establishment of patient care preferences and a clear plan for either mechanical ventilation or supportive care [23]. In our study, there was substantial variation in monitoring recommendations, with most recommending intermittent monitoring of observations and SpO_2_. Similarly, less than half the LHGDs specified criteria for medical review, treatment escalation or palliative care. While early warning scores are recommended in some oxygen guidelines to support recognition of clinical deterioration [30,32], recommendations regarding early warning scores and clinical deterioration systems varied widely across LHGDs.

A standardised core LHGD template for ward-based NIV could help reduce variation, allowing local adaptation to suit specific health service needs. In the UK, the BTS developed a national NIV audit tool to improve care, reduce morbidity and mortality, and track outcomes [20,43]. A similar approach could be adopted in Australia to support NIV implementation, evaluate patient outcomes, and assess clinician education and training. We propose piloting the new LHGD template across three health networks in metropolitan and regional health services in Victoria, auditing the NIV implementation and outcomes before and after the standardised LHGD implementation. This pilot will assess validity and feasibility before considering national rollout.

Although hospitals are required to develop LHGDs based on national and international clinical practice to support safe and consistent NIV implementation, our findings reveal significant variation in recommendations, many of which are not guideline-concordant. In the absence of clear local evidence-based guidance, NIV is often used less frequently and inconsistently across hospital settings [11,13]. Real-world practice also differs substantially from randomised trial evidence and guideline recommendations, with delays in initiation and limited monitoring contributing to lower success rates, higher failure rates, increased mortality, and uncertain efficacy in complex cases, particularly in ward environments [12,13,14,15,16,18,19,20]. In the UK national audits, the inpatient mortality dropped in 2019 to 26% from 34% in 2013, largely due to improvements in NIV implementation approaches, including patient selection, timely access to NIV and a respiratory nurse or physiotherapist providing specialist service leadership [19]. In Australia, currently, there are no national NIV audits; however, real-world ward-delivered NIV studies report in-hospital mortality for COPD at 11% [8], rising to 22% [44] for multifactorial respiratory failure. A prospective clinical audit of exacerbations of COPD hospital admissions in five states of Australia identified that 34% of eligible patients did not receive NIV, concluding that NIV is underutilised and may contribute to mortality [45]. These findings underscore an urgent need for local clinical guidance documents to be developed consistently based on guideline evidence and in a standardised manner to prevent the perpetuation of the well-documented global variation in NIV implementation and patient care. Importantly, there is a lack of evidence-based guidance regarding how NIV should be practically implemented in hospitals. Research and guidance are required to determine clinical roles (i.e., which disciplines can prescribe, initiate, and review NIV) and optimal ventilation parameters and monitoring strategies.

It is important to highlight that whilst this study found wide variation in the NIV LHDGs, it remains unknown how clinicians use LHDGs in practice, particularly clinicians who work in multiple health services. Prior research suggests such guidelines are often underutilised due to limited awareness and/or reliance on clinical experience [40,46]. Leveraging digital health records to embed LHGDs could enable real-time, automated clinical decision support for NIV [47]. Future research could investigate clinicians’ use and understanding of LHGDs in everyday practice to identify factors that hinder or facilitate their adoption. Additionally, conducting national audits of NIV utilisation and associated patient outcomes is essential for identifying clinical care targets that support improvements in quality and safety.

### Strengths and Limitations

This is the first study to evaluate acute NIV LHGDs to determine whether key recommendations align with clinical guidelines, offer sufficient technical detail to guide clinical decisions, and support effective implementation. The study employed a well-defined scoping review protocol to achieve these aims. The PROMPT database, an LHGD sharing platform accessed by over 100 health services across metropolitan, rural, and regional areas in Australia’s most populous states (population > 15 million), was used in this study. However, as not all services use PROMPT, the LHGDs identified may not fully represent all ward-based NIV guidance documents nationally. It is unlikely that including additional LHGDs would alter this result, given the significant variation between LHGDs.

## 5. Conclusions

There was substantial variation in the structure and content of LHGDs for ward-based NIV across Australian hospitals, including inconsistencies in initiation, monitoring, weaning, and detection of patient deterioration. Variation and limited alignment with major clinical guidelines may impact care quality and safety.

## Figures and Tables

**Table 1:** Recommended indication for NIV.

	Count (n)	Frequency (%)
Indications for NIV * -Acute exacerbation of COPD with hypercapnic respiratory failure -Acute pulmonary oedema -Neuromuscular/chest wall related disorders -Asthma -Obesity Hypoventilation Syndrome -Not specified -Symptom palliation	16 12 7 5 5 2 1	72.7 54.5 31.8 22.7 22.7 9.1 4.5
Fraction of inspired oxygen (%) to trigger NIV -Not specified -100 -55–60 -50 -40–50	17 2 1 1 1	77.3 9.1 4.5 4.5 4.5
pH and PaCO_2_ (ABG) definition to trigger NIV -PaCO_2_ or SaO_2_ definition not specified -pH < 7.35 & PaCO_2_ > 45 mmHg -pH < 7.30, with “high” PaCO_2_ -pH 7.2 to 7.35 with; PaCO_2_ > 45 mmHg, and, SaO_2_ < 90% -PaCO_2_ > 45 mmHg	11 8 1 1 1	50.0 36.4 4.5 4.5 4.5
Hypercapnia assessed with -Arterial blood gas -Venous blood gas -Not specified	17 3 2	77.3 13.6 9.1
SpO_2_ target (lower level of range) once NIV initiated * Not specified -85%+ -88%+ -90%+ -92%+	12 1 7 3 1	54.5 4.5 31.8 13.6 4.5
SpO_2_ target (higher level of range) once NIV initiated * -Not specified -92%+ -94%+ -96%+	13 7 4 1	59.1 31.8 18.2 4.5
Humidification with NIV	9	40.9
Advance care directive review	8	36.4
NIV communication with family and carers	8	36.4

* Categories are not mutually exclusive and cumulative percentages may sum to over 100. SpO_2_: Pulse oximetry oxygen saturation, SaO_2_: arterial oxygen saturation, PaCO_2_: partial pressure of carbon dioxide in arterial blood, ABG: Arterial blood gas, COPD: chronic obstructive pulmonary disease.

**Table 2:** Recommendations for NIV implementation and monitoring.

	At initiation of NIV		After Initiation of NIV	
	Count (n)	Frequency (%)	Count (n)	Frequency (%)
Ward location for NIV use * -General ward -Respiratory ward -Respiratory Care Unit -Not specified -General higher acuity area	12 5 4 3 1	54.5 22.7 18.2 13.6 4.5	N/A	N/A
ABG	17	77.3	17	77.3
VBG	3	13.6	2	9.1
SpO_2_ target range	9	40.9	13	59.1
Empirical initiation parameters * -IPAP -EPAP -BURR	12 12 5	54.5 54.5 22.7	N/A N/A N/A	N/A N/A N/A
Mask * -Oro Nasal -Nasal -Not stated -Nasal pillows	18 8 7 2	81.1 36.4 31.9 9.1	N/A N/A N/A N/A	N/A N/A N/A N/A
Humification	9	40.9	N/A	N/A
Ventilator alarms	9	40.9	N/A	N/A
Documentation of NIV settings * -Ventilator parameters (IPAP, EPAP, BURR) -Ventilator time used -Ventilator system (including mask type)	N/A	N/A	9 8 4	40.9 36.4 18.2
Documentation of patient’s clinical status * -Observations -ABG -SpO_2_ -Skin pressure injury -Nutrition status	N/A	N/A	21 19 13 13 5	95.5 86.4 59.1 59.1 22.7
Time until first observation -5–15 min -Not stated -30 min -60 min	N/A	N/A	15 4 2 1	68.2 18.2 9.1 4.5
Observation frequency -60 min -30 min -Not stated	N/A	N/A	10 8 4	45.4 36.4 18.2
Breaks from NIV -Not stated -Two hourly	N/A	N/A	13 9	59.1 40.9
NIV breaks periods -Not stated -Conventional oxygen -High nasal flow oxygen	N/A	N/A	18 3 1	81.8 13.6 4.5

* Categories are not mutually exclusive and cumulative percentages may sum to over 100. ABG: Arterial blood gas, VBG: Venous blood gas, IPAP: Inspiratory Positive Airway Pressure, EPAP: Expiratory Positive Airway Pressure, BURR: Back-up ventilation rates, SpO_2_: Pulse oximetry oxygen saturation, Respiratory Care Unit, NIV: Non-invasive ventilation.

**Table 3:** Recommendations for weaning NIV.

Characteristic	Count (n)	Frequency (%)
Maintain target SpO_2_ range -not specified -88–92%	9 13	40.9 59.1
Indications * -Not specified -Clinical improvement -Improved work of breathing and respiratory rate -Normalised PaCO_2_ -Normalised pH -Normalised oxygenation	15 5 4 3 3 2	68.2 22.7 18.2 13.6 13.6 9.1
Guidance -Not specified -Reduce time used—day -Reduce time use—day and night -Reduce pressure—day -As per medical officer -Reduce pressure—day and night	15 5 5 3 3 2	68.2 22.7 22.7 13.6 13.6 9.1
Guidance for NIV weaning for long-term NIV users	1	4.5
Guidance for referral for long-term NIV consideration	3	13.5

* Categories are not mutually exclusive and cumulative percentages may sum to over 100. NIV: Non-invasive ventilation.

**Table 4:** NIV LHGD template.

Document purpose	To provide structured clinical recommendations for ward-delivered management of NIV in adults with acute respiratory failure, encompassing initiation, monitoring, escalation, and weaning
Target audience	Clinicians (doctors, nurses and physiotherapists) trained in adult NIV implementation
Definitions	Acute hypercapnia as defined by ABG pH and PaCO_2_ values pH < 7.35 and PaCO_2_ ≥ 45 mmHg
Indications	NIV is the first-line treatment for patients with hypercapnic ARF in exacerbations COPD. * Consider implementing NIV in patients with other causes of hypercapnic ARF, e.g., in decompensated obesity hypoventilation syndrome or after extubation failure ABG should be used to determine the presence of hypercapnic ARF as defined above: Before initiating NIV, it is essential to consider and discuss treatment preferences and goals of care with patients and their carers. These discussions must be clearly documented for all patients. * Consideration for intermittent HFNO use when resting off NIV where local infrastructure allows
Contraindications	Normal oxygenation (without hypercapnia) * * There is a relative contraindication related to safety in the ward setting for NIV use in normal oxygen without hypercapnia in ARF associated with immunosuppressed or viral illness patients [21]. Dysfunctional breathing patterns (i.e., signs of respiratory muscle fatigue, increased work of breathing and paradoxical movements) Hypoxaemic ARF–consider HFNO or COT High severity of illness requiring urgent invasive mechanical ventilation. Facial burns, obstructed airway Active recurring nasal bleeding Altered conscious state (i.e., compromised airway protection or cooperation) (excluding reversible causes such as hypercapnia or severe fatigue) Surgical interventions-nasal and sinus (i.e., recent) Trauma of the maxillofacial (i.e., recent) Fractures–base of skull (including suspected)
Prescription	NIV prescription chart * *Integration into the digital health record is highly desirable* Target SpO_2_ range 88–92% in the setting of hypercapnia Should include the monitoring and maintenance (e.g., titration and weaning) to treatment aims
Initiation	ABG should be obtained prior to initiation—see indications ABG measurements are recommended before NIV commencement and as clinically indicated, especially in patients suspected hypercapnia due to the substantial limitations with SpO_2_ and venous blood gases Safe and effective initiation of NIV requires access to appropriate ventilators and consumables, supported by locally developed step-by-step guides and illustrative materials NIV device titration parameters (inspiratory and expiratory positive airway pressures, ventilation rate and oxygen flow for fraction of inspired oxygen litres) to maintain specified target SpO_2_ range: patients identified (or suspected *) with hypercapnia SpO_2_ 88–92% * *Consider conditions that increase the risk of hypercapnic respiratory failure, such as severe COPD, kyphoscoliosis, obesity hypoventilation, neuromuscular disorders, respiratory muscle weakness or a history of hypercapnia.* Clinical monitoring frequency should be guided by individual patient assessment and therapy goals. Documentation must include key physiological parameters: respiratory rate, heart rate, oxygen saturation, temperature, and blood pressure Initiation assessment and monitoring should occur every 15 min during the first hour of NIV therapy. An ABG should be obtained within 1–2 h of NIV initiation to assess the response to treatment. This frequency may be adjusted based on local resources, patient acuity, care goals, and clinical indication Assessment and ongoing management should document the respiratory rate, assessment of work of breathing, the NIV device used, any adjustments to ventilator pressure settings, and oxygen flow rate (L/min) to maintain target SpO_2_ levels as advised above
Maintenance	Observation intervals for physiological parameters should be tailored to the patient’s clinical status. As stability improves, monitoring frequency may decrease (e.g., from hourly to every four hours), depending on local service capacity Any change in observation frequency should be documented along with key physiological parameters—respiratory rate, work of breathing, heart rate, oxygen saturation, temperature, and details of the NIV device and oxygen flow rate used to maintain target SpO_2_ levels Clinical consideration for an ABG should be based on the individual patient assessment, including the frequency required. * Consider hydration and nutrition requirements during NIV implementation
Criteria for medical review	Physiological parameters changes including increased - Respiratory rate - NIV ventilator parameters (inspiratory and expiratory positive pressure or pressure support and positive end expiratory pressure, backup respiratory rate) - Supplemental oxygen flow rates (litres per minute) Increased ventilation parameter to meet the SpO_2_ target range Increased oxygen flow to meet the SpO_2_ target range Maximal oxygen flow to meet the SpO_2_ target range Unable to meet the SpO_2_ target range Clinical or patient/family concerns using NIV Other: As guided by the local early warning system, including hospital-specific alert systems
Escalation pathway	Adults with ARF receiving ward-based NIV are at risk of clinical deterioration and therefore require ongoing, regular assessment and monitoring. Early warning scores support clinical decision-making and guide escalation of care for deteriorating patients and should be available locally and integrated into the digital health record. [add in the local service clinical tool] i.e., modified early warning score (medical emergency team), including the referral mechanism to ICU (include how to contact the ICU junior and senior doctors, e.g., pager or mobile number) [add in the local work instruction] local ward upper limits for ward delivered NIV, i.e., device flow ventilator parameters or level of oxygen delivered
Weaning	Weaning is a key strategy for evaluating response to therapy and should be guided by individual patient assessment and clinical approach Weaning approach from NIV to be based in individual indications and response. * Consider using NIV during periods of high workload, such as during mobilisation, physiotherapy, rehabilitation and sleep Recommendations: - Maintain optimised ventilation parameters and wean daytime use hours first, leaving night use to support ventilation during sleep - Wean pressures as appropriate - Assess response to weaning with intermittent ABG - Continue to use COT or HFNO during rest periods off, maintain SpO_2_ target range When clinically indicated perform a room air assessment, including assessment of desaturation on COT, HFNO or room air (SpO_2_) episode (i.e., the time to desaturate), including the documentation of the essential physiological parameters of the respiratory and heart rate, oxygen saturation and temperature If patient is a known long-term NIV user –consideration for ongoing NIV requirement, i.e., wean to previous known ventilator settings and reassess. Provide a new revised NIV prescription for sleep periods (day and night) and for rest as indicated Referral pathway for known or suspected long-term NIV use (include contact method, e.g., medical staffpager or mobile) Upon cessation of NIV, patients should be transitioned to HFNO (if available) or COT, based on individual clinical need. Ongoing patient assessment and monitoring remain essential. It is recommended that the frequency of monitoring key physiological parameters—respiratory rate, heart rate, oxygen saturation, and temperature—be reduced to every four to six hours, or as clinically indicated.
NIV ward-delivered palliation	Clinical discretion is essential when using NIV to manage hypoxaemic or hypercapnic dyspnoea at the end of life. Patient preferences should be considered, as NIV may be poorly tolerated and can hinder communication Monitoring and escalation criteria are generally not applied to NIRS at end of life, where NIV is used for symptom relief. Adjustments or withdrawal should be considered once the patient is comfortable. Consider weaning to COT or HFNO devices (where available) as necessary, as most COT and HFNO devices are usually well tolerated, as the patients can communicate whilst using.
Associated documents	e.g., HFNO LHGD, COT LHGD, Escalation of care LHGD, NIV manufacturer instructions
References	e.g., BTS/ICS NIV guidelines [23], ERS/ATS NIV guidelines [21], TSANZ Acute Oxygen Position Statement [30], BTS Guideline for oxygen [32], ERS HFNO clinical practice guidelines [31]
Authors and review	Clinical staff positions and professional groups Review date Planned next review date Planned monitoring aligned to updated national and international guidelines

ABG: Arterial blood gas, ARF: Acute respiratory failure, COT: Conventional oxygen therapy, HFNO: High flow nasal oxygen, NIV: non-invasive ventilation, ICU: Intensive Care unit, LHGD: Local health guidance document, SpO_2_: Pulse oximetry oxygen saturation, pH: acid-base measurement.* Information to consider.

## Data Availability

The data used are not publicly available to maintain the anonymity of the health services that were included in the study.

## Supplementary Materials

The following supporting information can be downloaded at: https://www.mdpi.com/article/10.3390/jcm14228152/s1, Table S1: Further characteristics of included LHGD.
