# Supplementary material for: Non-Invasive Ventilation Therapy Implementation in Medical Wards—A Scoping Review to Understand Hospitals’ Protocols and Procedures
Source: J Clin Med. 2025 Nov 17;14(22):8152. doi: 10.3390/jcm14228152 (PMC12653429; doi:10.3390/jcm14228152)
Supplement: Supplementary file 1 [file jcm-14-08152-s001.zip › jcm-3944778-supplementary.pdf]

Supplementary Table S1: Further characteristics of included LHGD

	Count (n)	Frequency (%)
Document contributors*		
-Nursing staff	14	63.6
-Other	10	45.5
-Medical staff	8	36.4
-Physiotherapy staff	1	4.5
-Not specified	1	4.5
Year of #LHGD publication		
-2018 or prior	12	54.6
-2021-2022	1	4.5
-2019-2020	3	13.6
-Not specified	1	4.5
**-2022-2025**	5	22.7
Date of last review		
-2024-2025	5	22.7
2021-2023	8	36.4
-2019-2020	6	27.3
-Not specified	2	9.1
-2017 or prior	1	4.5
Document length (pages)		
-0-5	5	22.7
-6-10	10	45.5
-11-15	4	18.2
-16-20	3	13.6
LHGD objective stated	18	81.8
Definitions provided	16	72.7
Advantages of NIV stated	8	36.4

Contraindications to NIV*		
-Imminent cardiac or respiratory arrest	20	90.9
-Unable to protect own airway or manage secretions	17	77.3
-Untreated Pneumothorax	16	72.7
-Facial injury or burns	15	68.2
-Obstructed airway	12	54.5
-Requires intubation	10	45.4
-Altered conscious state	9	40.9
-Uncooperative/unable to tolerate mask	9	40.9
-Hypotension	8	36.3
-Uncontrolled vomiting/aspiration risk	7	31.8
-Severe refractory hypoxaemia	5	22.7
-Fractured base of skull	4	18.2
-Post-operative gastrointestinal or gastrointestinal bleed	4	18.2
-Raised intracranial pressure	4	18.2
-Not stated	3	13.6
-Transsphenoidal or oral surgery	3	13.6
-Severe asthma	2	9.1
-pH<7.25	2	9.1
-pH<7.15	1	4.5
-Oesophageal varices	1	4.5
-Bowel Obstruction	1	4.5
-Epistaxis	1	4.5
-Continuous nasogastric feeds	1	4.5
-Bronchopulmonary fistula	1	4.5
-Angioedema	1	4.5
Adverse effects of NIV*		
-Pressure sore	14	63.6
-Aerophagia	12	54.5
-Not stated	8	36.3
-Hypotension	6	27.3
-Barotrauma/Pneumothorax	6	27.3
-Claustrophobia	5	22.7
-Aspiration	5	22.7
-Eye irritation/Ear pain	4	18.2
NIV systems component specified		
- Interface (e.g., masks)	16	72.7
-Ventilator (e.g., brand and model)	14	63.6
-Diagram	4	18.2
Guidance for NIV Mask fit*		
-Fit checklist	12	54.5
-Size guide	7	31.8
-Not stated	6	27.3
-Troubleshoot	4	18.2
-Fit tightly	3	13.6
Guidance for patient positioning	15	57.7

**Categories are not mutually exclusive and cumulative percentages may sum to over 100*
NIV: Non-invasive ventilation
